# The complete plastid genome sequence of *Phyllodium pulchellum* (L.) Desv. (Leguminosae)

**DOI:** 10.1080/23802359.2019.1693299

**Published:** 2019-11-22

**Authors:** Mingsong Wu, Ming Qin, Xin Qian, Kai Zhang, Tieyao Tu

**Affiliations:** a Key Laboratory of Plant Resources Conservation and Sustainable Utilization, South China Botanical Garden, Chinese Academy of Sciences, Guangzhou, China;; b University of Chinese Academy of Sciences, Beijing, China

**Keywords:** Desmodieae, legume, medicinal plant, papilionoideae, phylogeny

## Abstract

This study investigated the complete plastid genome of *Phyllodium pulchellum*, which represents the first report of the complete plastome for the genus *Phyllodium* in the tribe Desmodieae of the subfamily Papilionoideae. De novo assembly and annotation showed that the plastid genome is a typical quadripartite structure consisting of two inverted repeats (IR = 24,286 bp), one small single copy (SSC = 18,334 bp), and one large single copy (LSC = 82,715 bp). We found 111 unique genes, including 77 protein-coding genes, 30 tRNA genes, and 4 rRNA genes. Phylogenetic analysis based on the newly sequenced plastid genome of *Ph. pulchellum* and 11 plastomes obtained from GenBank recovered a strongly supported phylogenetic backbone of the tribe Desmodieae and a sister-relationship between *Phyllodium* and *Ohwia*.

*Phyllodium pulchellum* (L.) Desv., a small shrub of the legume family with branchlets equipped with white or gray pubescence and five or six flowers, enclosed by a pair of orbicular leaflike bracts, is well-known as a traditional medicine against liver fibrosis, rheumatoid arthritis pain, swelling, fever, malaria, and even eye diseases (Rahman et al. 2013; Velmurugan et al. 2014). Herein we report the first complete plastid genome of *Ph. pulchellum*, aiming at evaluating the utilities of plastome in the reconstruction of the phylogenetic position of the species in the tribe Desmodieae of the subfamily Papilionoideae.

Fresh leaves of *Ph. pulchellum* were collected from Huangmao Island of Zhuhai, China (N 22°2′30.63″, E 113°39′42.40″). The voucher specimen (Mingsong Wu, WuMS224) was deposited in the herbarium of South China Botanical Garden (IBSC). Total genomic DNA was extracted using a modified CTAB method (Doyle and Doyle 1987). Paired-end (PE) sequencing was completed on the Illumina HiSeq X-Teninstrument at Beijing Genomics Institute (BGI) in Wuhan, China. In total, 3.23 Gb of clean data were generated for assembling the complete plastome using the GetOrganelle pipeline (Jin et al. 2018). The plastid genome sequence of *Ohwia caudata* (Thunb.) H. Ohashi (NC_044105) was used as a reference to annotate the plastome using the GeSeq (Tillich et al. 2017). Geneious R11 (Kearse et al. 2012) was used to check the accuracy of the assembly, and to adjust the start/stop codons and intron/exon boundaries of the annotation. The annotated plastome was deposited in GenBank under the accession number MN614126.

The complete plastid genome of *Ph. pulchellum* is 149,620 bp in length, with 35.1% GC content, and contains a typical quadripartite plastid structure with two inverted repeats (24,286 bp) separated by a large single-copy region (82,715 bp) and a small single-copy region (18,334 bp). We found 111 unique genes, including 77 protein-coding genes, 30 tRNA genes, and 4 rRNA genes.

To reveal the phylogenetic position of *Ph. pulchellum* within the tribe Desmodieae, we downloaded 11 complete plastid genome sequences from the GenBank, of which three species in the tribe Phaseoleae were used as outgroups. We used the MAFFT v.7.308 (Katoh and Standley 2013) to align the data matrix with default parameters. We constructed the maximum-likelihood (ML) phylogenetic tree using RAxML v.8.2.10 (Stamatakis 2014) under the GTR + G substitution model as suggested by ModelFinder (Kalyaanamoorthy et al. 2017). Our phylogenetic analysis strongly suggested that *Ph. pulchellum* is closely related to *Ohwia caudata* with 100% bootstrap support (Figure 1). The backbone of Desmodieae including nine species from seven genera is well resolved with strong supports. Our study suggested that the complete plastome sequences could be powerful in reconstructing the phylogenetic relationships among *Ph. pulchellum* and related species in Desmodieae when a more comprehensive sampling is available in the future.

**Figure 1.**
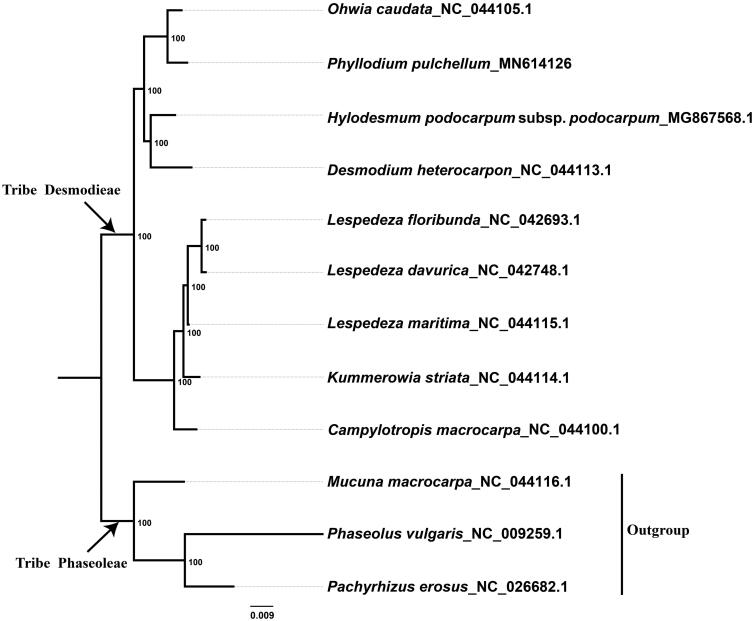
The maximum-likelihood (ML) phylogenetic tree based on the complete plastid genome sequences. Numbers at the right of nodes are bootstrap support values.
